# The Activity-Induced Long Non-Coding RNA *Meg3* Modulates AMPA Receptor Surface Expression in Primary Cortical Neurons

**DOI:** 10.3389/fncel.2017.00124

**Published:** 2017-05-03

**Authors:** Men C. Tan, Jocelyn Widagdo, Yu Q. Chau, Tianyi Zhu, Justin J.-L. Wong, Allen Cheung, Victor Anggono

**Affiliations:** ^1^Clem Jones Centre for Ageing Dementia Research, The University of QueenslandBrisbane, QLD, Australia; ^2^Queensland Brain Institute, The University of QueenslandBrisbane, QLD, Australia; ^3^Gene and Stem Cell Therapy Program, Centenary InstituteSydney, NSW, Australia; ^4^Sydney Medical School, University of SydneySydney, NSW, Australia

**Keywords:** long non-coding RNA, AMPA receptors, receptor trafficking, maternally expressed gene 3 (*Meg3*), PTEN/PI3K/AKT, synaptic potentiation, LTP, epigenetics

## Abstract

Transcription of new RNA is crucial for maintaining synaptic plasticity, learning and memory. Although the importance of synaptic plasticity-related messenger RNAs (mRNAs) is well established, the role of a large group of long non-coding RNAs (lncRNAs) in long-term potentiation (LTP) is not known. In this study, we demonstrated the expression of a lncRNA cluster, namely maternally expressed gene 3 (*Meg3*), retrotransposon-like gene 1-anti-sense (*Rtl1-AS*), *Meg8* and *Meg9*, which is located in the maternally imprinted *Dlk1-Dio3* region on mouse chromosome 12qF1, in primary cortical neurons following glycine stimulation in an N-Methyl-D-aspartate receptor (NMDAR)-dependent manner. Importantly, we also validated the expression of *Meg3*, *Meg8* and *Meg9* in the hippocampus of mice following cued fear conditioning *in vivo*. Interestingly, *Meg3* is the only lncRNA that is expressed in the nucleus and cytoplasm. Further analysis revealed that *Meg3* loss of function blocked the glycine-induced increase of the GluA1 subunit of AMPA receptors on the plasma membrane, a major hallmark of LTP. This aberrant trafficking of AMPA receptors correlated with the dysregulation of the phosphatidylinoside-3-kinase (PI3K)/AKT signaling pathway and the downregulation of the lipid phosphatase and tensin homolog (PTEN). These findings provide the first evidence for a functional role of the lncRNA *Meg3* in the intricate regulation of the PTEN/PI3K/AKT signaling cascade during synaptic plasticity in neurons.

## Introduction

The ability of neurons to modulate the strength of their connectivity, a process known as synaptic plasticity, has long been postulated as a cellular mechanism of learning and memory (Nicoll, 2017). One of the best-studied forms of such plasticity is long-term potentiation (LTP) which produces a persistent increase in the synaptic strength in neurons, characterized by an increase in the size of dendritic spines and the number of synaptic AMPA-type glutamate receptors (α-amino-3-hydroxy-5-methyl-4-isoxazolepropionic acid receptors (AMPARs)). A prominent theory of LTP and long-term memory holds that new messenger RNAs (mRNAs) must be transcribed and new proteins must be synthesized in order to maintain the long-lasting increase in the magnitude of synaptic response and memory consolidation (Huang et al., 1996; Adams and Dudek, 2005). Initial studies have shown that the inhibition of transcription and translation block the synthesis of new AMPARs and late-phase LTP in acute hippocampal slices (Frey et al., 1993; Nguyen et al., 1994; Nayak et al., 1998). More importantly, they also block the maintenance of LTP and long-term memory retention *in vivo* (Frey et al., 1996; Kandel, 2001; Alberini, 2009; Radwanska et al., 2011; Hagena and Manahan-Vaughan, 2013). Interestingly, commonly used transcription inhibitors such as actinomycin D prevent not only transcription of new mRNAs, but all RNA polymerase II-dependent transcription, which includes various forms of non-coding RNAs.

Long non-coding RNAs (lncRNAs), broadly classified as being more than 200 nucleotides in size, represent a large proportion of the transcriptome and are widely expressed in the mammalian brain in a cell type- and developmental stage-specific manner (Mercer et al., 2008), but a vast proportion are still functionally uncharacterized. In the brain, a number of lncRNAs have been shown to play prominent roles during neural development and differentiation (Bond et al., 2009; Bernard et al., 2010; Roberts et al., 2014), and they are increasingly associated with human neurological disorders (Briggs et al., 2015). However, the role of lncRNAs in post-mitotic neurons is not known. Mechanistically, lncRNAs are capable of binding DNA, RNA and proteins, allowing them to perform a sensory, guiding or scaffolding function and influence multiple processes, from transcriptional regulation to the modulation of protein activity (Mercer and Mattick, 2013). The versatility and dynamics of lncRNAs are highly suited for the rapid neuronal response to extracellular signals, synaptic plasticity and adaptive behaviors. However, despite some evidence of stimulus-dependent expression of neuronal lncRNAs (Briggs et al., 2015; Maag et al., 2015), there is currently no empirical support for lncRNA function in synaptic plasticity or the regulation of learning and memory.

The *Dlk1-Dio3* imprinted region, located on mouse distal chromosome 12qF1 (or human chromosome 14q32), contains a cluster of lncRNA genes that are selectively expressed from the maternally inherited allele, namely maternally expressed gene 3 (*Meg3*), retrotransposon-like gene 1-anti-sense (*Rtl1-AS*), *Meg8* and *Meg9* (da Rocha et al., 2008). *Meg3*, also known as gene trap locus 2 (*Gtl2*), is highly expressed in neurons in the forebrain of both developing and adult mice (McLaughlin et al., 2006; Qu et al., 2013). However, genetic deletion resulting in the loss of *Meg3* from the maternal allele causes perinatal death in mice (Zhou et al., 2010), precluding further observation of any potential neurological phenotypes. The cellular function of *Meg3* in regulating cell death and differentiation has been well studied, and it is thought to play a role as a tumor suppressor as its expression is often markedly downregulated in various types of cancers (Zhou et al., 2012). However, despite being highly expressed in the brain, *Meg3* currently has no known function in post-mitotic neurons.

In the present study, we reveal that the expression of all four lncRNAs within the *Dlk1-Dio3* imprinted locus is dynamically upregulated in primary cultured neurons following glycine stimulation, a validated method of chemically inducing N-Methyl-D-aspartate receptor (NMDAR)-dependent LTP *in vitro* (Lu et al., 2001). Importantly, increased expression of these lncRNAs can also be observed in the hippocampus of mice that undergo a fear-conditioned associative learning paradigm. Short hairpin RNA (shRNA)- and antisense oligonucleotide (ASO)-mediated knockdown of *Meg3*, the transcript of which is present in both the nucleus and cytoplasm, enhances surface AMPAR expression under basal conditions and prevents a further increase in the expression of these receptors on the plasma membrane following glycine stimulation. These effects are associated with an overall activation of the phosphatidylinoside-3-kinase (PI3K)/AKT signaling pathway, which plays a major role in the activity-dependent delivery of AMPARs to the plasma membrane. These results provide insights into the role of Meg3 in modulating the PI3K/AKT/phosphatase and tensin homolog (PTEN) signaling pathway in order to fine-tune the level of surface AMPARs during synaptic plasticity.

## Materials and Methods

### Animals

Adult male C57BL/6 mice (8–12 weeks old) were used for the fear-conditioning experiments, whereas embryonic day 17 mice were used for primary neuronal cultures. Mice were housed on a 12 h light/dark schedule, and fed *ad libitum*. This study was carried out in accordance with the recommendations of Australian code for the care and use of animals for scientific purposes by the National Health and Medical Research Council. All testing was conducted during the light phase in red light-illuminated rooms following protocols approved by the University of Queensland Animal Ethics Committee.

### Antibodies

Specific antibodies against CREB (cAMP response element binding protein, 86B10), phosphorylated CREB (Ser133; 87G3), ERK1/2 (extracellular signal-regulated kinase 1/2, 137F5), phosphorylated ERK (Thr202/Tyr204; E10), AKT (C67E7), phosphorylated AKT (Thr308; D25E6), phosphorylated AKT (Ser473; D9E), p85 (19H8), phosphorylated p85 (Tyr458; 4288), PTEN (D4.3), β-actin (13E5) and GAPDH (14C10) were purchased from Cell Signaling Technology. The GluA1 (MAB2263) and phosphorylated GluA1 (Ser831; AB5847) antibodies were from Millipore.

### Primary Neuronal Cultures

Cortical neurons were prepared and maintained in Neurobasal medium containing 2% B27, 2 mM Glutamax, 50 U/ml penicillin, 50 μg/ml streptomycin, and 1% fetal bovine serum as described previously (Anggono et al., 2013; Widagdo et al., 2015). All reagents were obtained from Invitrogen.

### Glycine-Induced Chem-LTP

**Figure 1:**
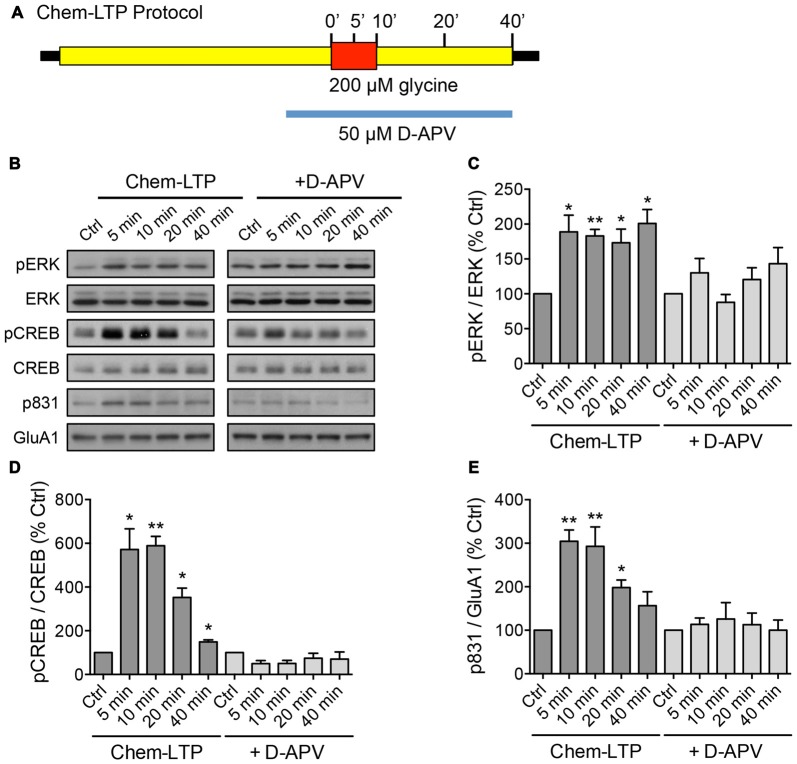
**Glycine induces the phosphorylation of extracellular signal-regulated kinase (ERK), cAMP response element binding protein (CREB) and GluA1 in mouse cortical neurons. (A)** Schematic diagram of the glycine-induced chem-long-term potentiation (LTP) protocol performed in days *in vitro* (DIV) 15–17 neurons. The N-Methyl-D-aspartate receptor (NMDAR)-dependent effect was determined by including the NMDAR antagonist, D-APV in the treatment. Protein lysates were collected by direct lysis in SDS sample buffer at different time points as indicated in the diagram. **(B–E)** Western blot analyses of phosphorylated CREB (Ser133), total CREB, phosphorylated ERK (Thr202/Tyr204), total ERK, phosphorylated GluA1 (Ser831) and total GluA1 levels during chem-LTP in the presence or absence of the NMDAR antagonist, D-APV. Quantitation of pCREB/CREB, pERK/ERK and p831/GluA1 was normalized to the 0 min control in each set. Data represent the mean ± SEM of four independent experiments (*n* = 4, one-way ANOVA, *P < 0.05, **P < 0.01).

Chem-LTP was performed on mouse cortical neurons at days *in vitro* (DIV) 15–17 as previously described, with slight modifications (Hussain et al., 2014). Briefly, neurons were incubated for 1 h in artificial cerebrospinal fluid (ACSF; 125 mM NaCl, 2.5 mM KCl, 1.5 mM CaCl_2_, 25 mM HEPES, pH 7.4, 33 mM glucose, 1 mM MgCl_2_, 500 nM tetrodotoxin (Tocris), 20 μM bicuculline (Tocris), 1 μM strychnine (Sigma)) prior to 10-min incubation with 200 μM glycine in magnesium-free ACSF to induce chem-LTP (Figure 1A). For the 20 and 40 min time points, neurons were recovered in ACSF for another 10 or 30 min prior to lysis. To block NMDAR activity, 50 μM D-APV (Tocris) was added to the ACSF 10 min prior to the induction of LTP, and remained present throughout the treatment.

### RNA Extraction and Quantitative Real-Time PCR (qPCR)

Total RNA was extracted with the Directzol RNA MiniPrep kit (Zymo Research), including on-column DNAse treatment. For nuclear and cytoplasmic fractions, cortical neurons were lyzed in RLN buffer (50 mM Tris-HCl pH 8.0, 140 mM NaCl, 1.5 mM MgCl_2_, 0.5% Nonidet P-40, 1000 U/ml RNaseOUT, 1 mM DTT) as previously described (Djebali et al., 2012), with slight modifications. The lysate was spun at 300× *g* for 3 min at 4°C to pellet the nuclei. RNA from the supernatant (cytoplasm) and the nuclear pellet was extracted using the Directzol kit (Zymo) by adding 6–7 volumes of TRI reagent. For the nuclear pellet, the sample was further passed through a syringe needle. Equal amounts of RNA were reverse transcribed using the QuantiTect Reverse Transcription kit (Qiagen). Quantitative real-time polymerase chain reaction (qPCR) analysis was performed using the QuantiFast SYBR green kit (Qiagen) in a CFX96/384 Touch Real-Time PCR system (Bio-Rad). Gene expression was calculated using the standard comparative Ct method after normalizing to the housekeeping gene, *Actb* or *GapDH*. The sequences for the primers used were as follows: *Meg3* (Forward: 5′-GGGAGCAGCTATGGATCACC-3′; Reverse: 5′-ATAGCGCCCCCTATTCATGC-3′), *Rtl1-AS* (Forward: 5′-TAGACCAGGACCTCTCTGCC-3′; Reverse: 5′-GCGGCATGGTTCACAAAACT-3′), *Meg8* (Forward: 5′-GCAGAACCACTGGTGGAAGTT-3′; Reverse: 5′-GTGTCCCGTGTCCACAATAG-3′), *Meg9* (Forward: 5′-AGGCTATCACCATCCCCCTT-3′; Reverse: 5′-TCCTAGACCTTGCCCGATGA-3′), *Actb* (Forward: 5′-CGGTTCCGATGCCCTGAGGCTCTT-3′; Reverse: 5′-CGTCACACTTCATGATGGAATTGA-3′), *GapDH* (Forward: 5′-TGCCCCCATGTTTGTGATG-3′; Reverse: 5′-TGTGGTCATGAGCCCTTCC-3′), *Egr1* (Forward: 5′-GCCTTCGCTCACTCCACTAT-3′; Reverse: 5′-CTGGGTTTGATGAGCTGGGA-3′ *Gria2* (Forward: 5′-CGAGGGGATATTTTGTGGATGC-3′; Reverse: 5′-ACTGAACCATCCCTACCCGAAA-3′), *U6* (Forward: 5′-TCGCTTCGGCAGCACATATAC-3′; Reverse: 5′-TTCACGAATTTGCGTGTCATCC-3′).

### Fear Conditioning Paradigm

Two-month old male mice were subjected to a cued fear conditioning paradigm. The “paired” group received five pairings of 2 min 80 dB white noise as the conditioned stimulus (CS), co-terminated with a 1 s 0.7 mA footshock as the unconditioned stimulus (US). The “unpaired” group was exposed to the same CS and US but with randomly applied footshock. The “context” group received the CS only and the “naïve” group comprised home-caged mates that did not undergo training. All animals were returned to their cages prior to being sacrificed.

### Lentiviral-Mediated Knockdown of *Meg3*

Two shRNA sequences targeting mouse *Meg3* (sh#1: 5′-ACAAGATGCTTACAGAAATAC-3′; sh#2: 5′-TGAGTGATAGACTACATATAT-3′) were cloned into an FG12 plasmid containing a GFP cassette, and packaged into HEK293 cells as previously described (Widagdo et al., 2016). Neurons were transduced with lentivirus between DIV 8–11 for 6 h, after which they were further incubated for 5 days prior to harvesting or conducting the chem-LTP assay. The efficacy of the transduction was assessed by the GFP expression of neurons, which was typically >90%.

### ASO-Mediated Knockdown of *Meg3*

Neurons were incubated with 1 μM ASO between DIV 12–14 for 48 h prior to harvesting or conducting the chem-LTP assay. The ASOs were 20 nucleotides in length, wherein the central gap segment comprising 10 2′-deoxynucleosides was flanked on the 5′ and 3′ ends by five 2′-*O*-methoxyethyl-modified nucleosides. All internucleoside linkages were phosphorothioate linkages. The sequences of the ASOs were as follows: control ASO: 5′-CCUUCCCTGAAGGTTCCUCC-3′ (Graham et al., 2007); *Meg3* ASOs: a mixture of two *Meg3* targeting sequences (ASO-1: 5′-AACAGCAAATGGCACAGGAA-3′, ASO2: 5′-UUAUUTATGGACCTCAGGUG-3′).

### Surface Biotinylation Assay

The chemical labeling of surface proteins with biotin was performed as described previously (Anggono et al., 2011). Briefly, neurons were washed twice with ACSF and incubated with 0.5 mg/ml Sulfo-NHS-SS-Biotin (Pierce) for 30 min on ice. Free biotin was quenched by washing the cells twice with ice-cold 50 mM glycine (pH 7.4 in ACSF). Cultures were lyzed and sonicated in RIPA buffer (1% Triton X-100, 0.5% Na-deoxycholate, 0.1% SDS, 2 mM EDTA, 2 mM EGTA, 50 mM NaF, 10 mM Na-pyrophosphate in Tris-buffered saline) and incubated with Neutravidin beads (Pierce) for 3 h at 4°C. Beads were washed three times, eluted with 2× SDS sample buffer, then analyzed by western blotting.

## Results

### Activity-Dependent Upregulation of lncRNAs within the *Dlk1-Dio3* Locus *In Vitro* and *In Vivo*

**Figure 2:**
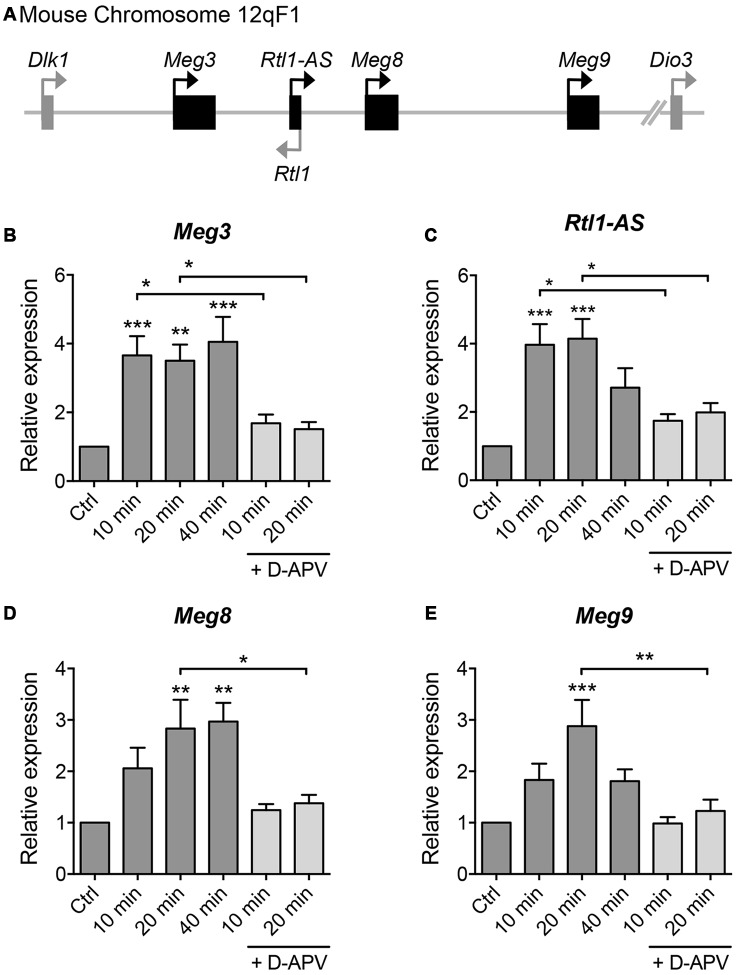
**Glycine stimulation induces the expression of long non-coding RNAs (lncRNAs) within the *Dlk-Dio3* imprinted locus in an NMDAR-dependent manner. (A)** Schematic representation of *Dlk1-Dio3* locus in the mouse chromosome 12qF1, which contains a cluster of maternally expressed lncRNAs (black rectangle). **(B–E)** Quantitative real-time polymerase chain reaction (qPCR) analyses of maternally expressed gene 3 (*Meg3*), retrotransposon-like gene 1-anti-sense (*Rtl1-AS*), *Meg8* and *Meg9* in primary neurons following glycine stimulation, after normalization with *Actb*. Data represent the mean ± SEM of at least nine independent experiments (*n* = 9–11, one-way ANOVA, *P < 0.05, **P < 0.01, ***P < 0.001).

To screen for lncRNAs which may play a role in synaptic plasticity, we first established an *in vitro* model of chemically induced LTP (chem-LTP) in primary cultured neurons (Figure 1A). Mouse cortical neurons were treated with 200 μM glycine, an NMDAR co-agonist, for 10 min, a protocol that is known to induce NMDAR-dependent insertion of new AMPARs and enhance network activity, resulting in a long-lasting strengthening of excitatory neurotransmission (Lu et al., 2001). This chem-LTP protocol caused a robust and persistent activation of the mitogen-activated protein kinase signaling cascade, as shown by a robust increase in ERK1/2 phosphorylation at Thr-202 and Tyr-204 (Figures 1B,C). In addition, glycine stimulation also led to enhanced phosphorylation of the transcription factor CREB and the GluA1 subunit of AMPARs at Ser-133 and Ser-831, respectively (Figures 1B,D,E), all of which are biochemical hallmarks of LTP (Ehlers, 2003). Glycine-induced increases in ERK, CREB and GluA1 phosphorylation were effectively blocked by D-APV, a selective NMDAR antagonist (Figures 1B–E). Using this chem-LTP paradigm, we profiled the expression levels of well-annotated lncRNAs in mouse primary neurons by qPCR. Unexpectedly, we detected a concurrent upregulation of lncRNAs located within the maternally imprinted *Dlk1-Dio3* locus, namely *Meg3*, *Rtl1-AS*, *Meg8* and *Meg9* (Figures 2A–E). In contrast, the mRNA expression of *Gria2* was not altered by glycine stimulation (Supplementary Figure S1), suggesting that the response profile for *Meg3*, *Rtl1-AS*, *Meg8* and *Meg9* was indeed target specific. Moreover, the application of D-APV abolished glycine-induced expression of these lncRNAs, suggesting that their rapid induction is dependent on NMDAR activation (Figures 2B–E).

**Figure 3:**
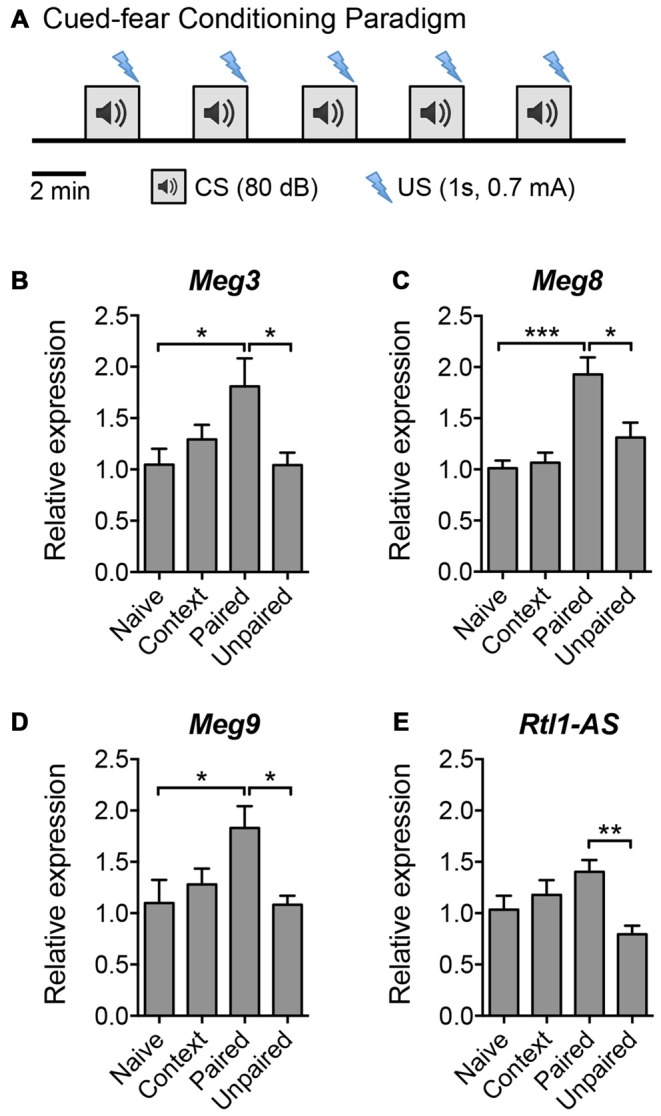
**The expression of *Dlk1-Dio3* imprinted lncRNAs in the mouse hippocampus following cued fear conditioning. (A)** Schematic representation of the cued fear conditioning paradigm consisting of five pairings of the conditioned stimulus (CS, white noise) and the unconditioned stimulus (US, footshock). The US was randomized in the unpaired protocol. Mice were sacrificed 2 h post-training for hippocampal tissue isolation and RNA extraction. **(B–E)** qPCR analyses of *Meg3*, *Meg8, Meg9* and *Rtl1-AS* expression after normalization with *Actb*. Data represent the mean ± SEM (*n* = 5–6, one-way ANOVA, *P < 0.05, **P < 0.01, ***P < 0.001).

Given that LTP plays an important role in learning and memory, we next evaluated the expression of the aforementioned maternally imprinted lncRNAs in response to a learning paradigm *in vivo*. We performed a cued fear conditioning assay and analyzed the lncRNA expression levels in the hippocampus of mice that: (a) had not been exposed to the training protocol (naïve); (b) were exposed to the cage and tone without receiving foot shocks (context); (c) were subjected to foot shocks that co-terminated with the tone (paired); or (d) received random shocks in the same context (unpaired) (Figure 3A). qPCR analysis revealed that the expression levels of *Meg3*, *Meg8* and *Meg9* were significantly upregulated in mice subjected to the paired protocol compared to naïve animals, or those exposed to random shocks or context only (Figures 3B–D). The expression of *Rtl1-AS* exhibited a trend for upregulation in the paired animals but did not reach statistical significance when compared to the level in naïve mice (Figure 3E). These results suggest that among the *Dlk1-Dio3* lncRNAs, the expression levels of at least *Meg3*, *Meg8* and *Meg9* are elevated during associative learning *in vivo*. Interestingly, when examined 24 h post-training, the expression of *Meg9* remained high although it did not reach statistical significance, whereas the expression of *Meg3* was significantly downregulated (Supplementary Figure S2). Collectively, these data show that the expression of the *Dlk1-Dio3* derived lncRNAs is dynamically regulated by neuronal activity both *in vitro* and *in vivo*.

### Loss of *Meg3* Expression Inhibits Glycine-Induced Increase in Surface GluA1

**Figure 4:**
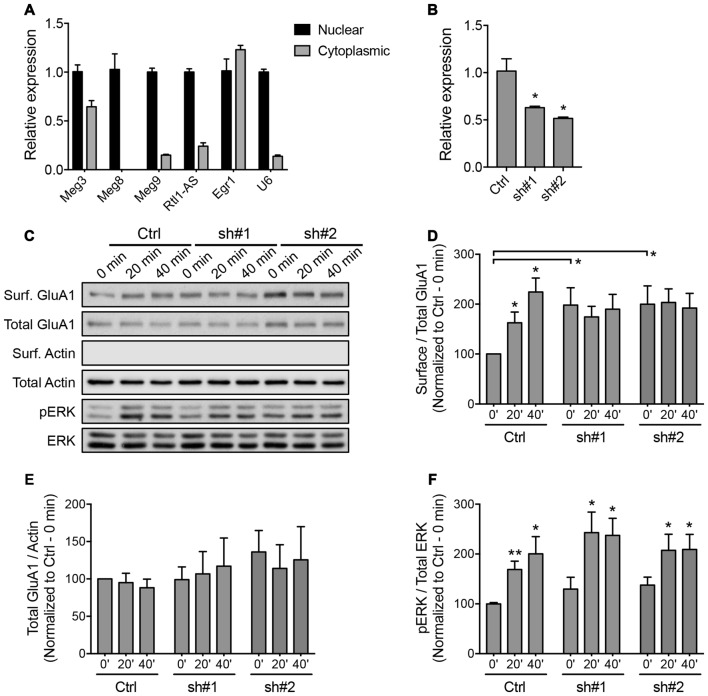
**Downregulation of *Meg3* disrupts α-amino-3-hydroxy-5-methyl-4-isoxazolepropionic acid receptor (AMPAR) trafficking in primary neurons. (A)** qPCR analysis of the *Dlk1-Dio3* lncRNAs in the nuclear and cytoplasmic fractions of cortical neurons (*n* = 3). **(B)** The efficiency of lentiviral mediated knockdown of *Meg3* in cortical neurons using two independent short hairpins RNA (shRNAs) or the FG12 control vector was quantified by qPCR analysis (*n* = 3, unpaired *t*-test). **(C)** Surface biotinylation assay performed in *Meg3* knockdown and control neurons following chem-LTP. Representative western blots show the protein levels in the surface and total fractions. The absence of β-actin in the surface fractions was used to validate the biotinylation assay. **(D,E)** The effects of *Meg3* knockdown on the levels of surface and total GluA1 expression were quantified as **(D)** surface/total receptor ratio and **(E)** total receptor/β-actin ratios, respectively, and normalized to the 0 min time point of the control cells. Data represent the mean ± SEM of three independent experiments (*n* = 4–5, two-way ANOVA, *P < 0.05). **(F)** The effect of *Meg3* knockdown on the activation of ERK during chem-LTP was quantified by measuring the ratios of phosphorylated over total ERK (*n* = 8–9, two-way ANOVA, *P < 0.05, **P < 0.01).

The majority of lncRNAs reside in the nucleus and associate with chromatin to perform epigenetic-related functions. The *Dlk1-Dio3*-derived lncRNAs have been shown to interact with the Polycomb Repressive Complex 2 (PRC2) chromatin modifying complex (Zhao et al., 2010; Kaneko et al., 2014). We performed a biochemical nuclear/cytoplasmic fractionation assay and confirmed that all of the *Dlk1-Dio3* lncRNAs were highly enriched in the nucleus, with the exception of *Meg3*, approximately one-third of which was found in the cytoplasmic fraction (Figure 4A). qPCR analysis revealed a proportional increase in the expression of nuclear and cytoplasmic *Meg3* in neurons that underwent chem-LTP stimulation (Supplementary Figure S3A). Intrigued by the fact that *Meg3* may play a role outside the nucleus, we focused on its neuronal function in subsequent experiments.

To evaluate the functional importance of *Meg3* in primary neurons, we designed two independent shRNAs targeting the exonic region of the *Meg3* sequence. Cortical neurons transduced with lentiviral particles expressing these two *Meg3* shRNAs exhibited a significant reduction in *Meg3* expression compared to neurons that were transduced with control lentiviral particles (Figure 4B). A similar reduction in *Meg3* expression was also observed in both the nuclear and cytoplasmic fractions (Supplementary Figure S3B). Using the shRNA-mediated knockdown approach, we analyzed the effect of *Meg3* loss of function on chem-LTP by examining the levels of AMPAR expression on the plasma membrane. Surface biotinylation assays revealed a persistent increase in the surface expression of the GluA1 subunit of AMPARs up to 40 min post-chem-LTP induction (Figures 4C,D), consistent with the central dogma that the persistent increase in the number of surface AMPARs supports the induction and maintenance of LTP. Surprisingly, *Meg3* knockdown blocked the glycine-induced increase in surface GluA1, which seemed to be occluded due to an elevated level of GluA1 on the plasma membrane under basal conditions (Figures 4C,D). Interestingly, *Meg3* knockdown did not have significant effects on the expression of total GluA1 proteins or NMDAR-dependent activation of the ERK signaling pathway (Figures 4C,E,F). Similar observations were made using the ASO-mediated knockdown approach (Supplementary Figure S4). Collectively, these data suggest that the aberrant levels of surface GluA1 are likely due to impaired AMPAR trafficking.

### Loss of *Meg3* Expression Dysregulates the PTEN/PI3K/AKT Signaling Pathway

**Figure 5:**
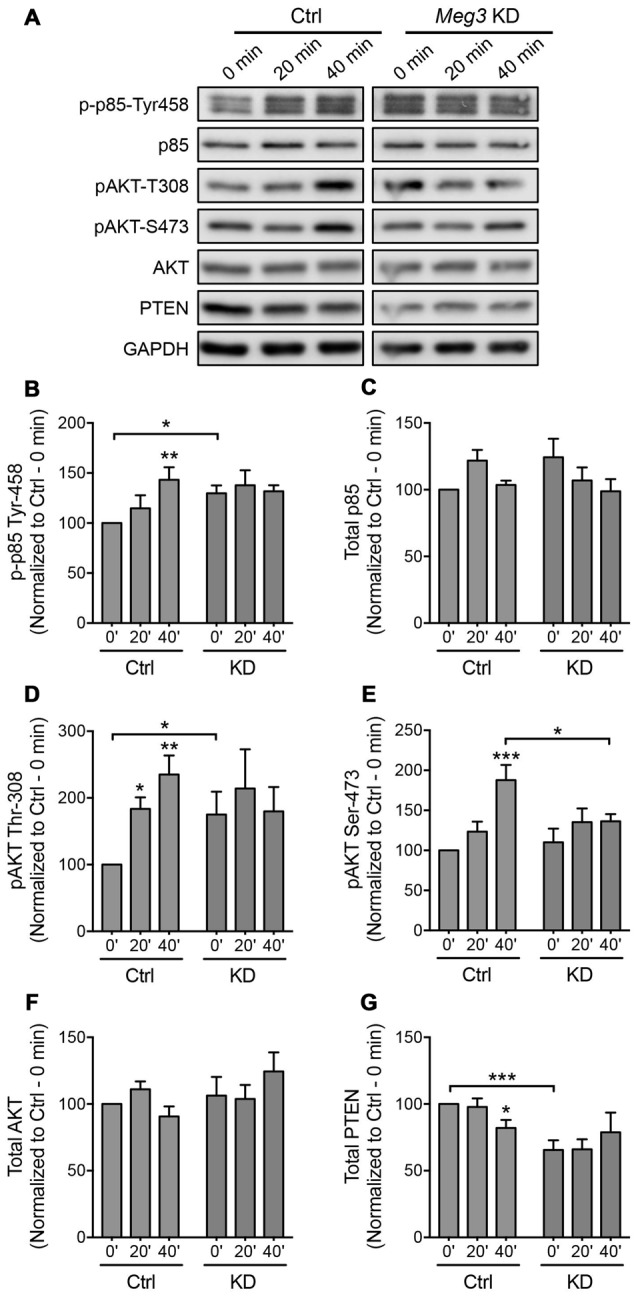
***Meg3* knockdown dysregulates phosphatase and tensin homolog (PTEN)/phosphatidylinoside-3-kinase (PI3K)/AKT signaling during chem-LTP. (A)** Cortical neurons were transduced with either *Meg3* shRNA#2 or FG12 control lentiviral particles for 5 days and subjected to chem-LTP assay. Protein lysates were analyzed by western blotting. **(B–G)** Activation of the p85 **(B)** and AKT **(D,E)** pathways during chem-LTP was measured by quantifying the levels of p85 (Tyr-458) and AKT (Thr-308 and Ser-473) phosphorylation over total protein expression. The relative expression of total p85 **(C)** AKT **(F)** and PTEN **(G)** normalized to GAPDH in control and *Meg3* knockdown neurons. Data represent the mean ± SEM of four independent experiments (*n* = 7, two-way ANOVA, *P < 0.05, **P < 0.01, ***P < 0.001).

We next sought to identify an upstream regulator of AMPAR insertion, which has previously been linked to *Meg3* in other cellular systems. Several recent studies have demonstrated a crosstalk between *Meg3* and the PI3K/AKT pathway. In macrophages and endothelial cells, the downregulation of *Meg3* leads to the activation of PI3K/AKT signaling through an unknown mechanism (Pawar et al., 2016; Qiu et al., 2016). In neurons, the PI3K/AKT pathway plays an important role in neuronal survival, synaptic plasticity, learning and memory (Sanna et al., 2002; Man et al., 2003; Opazo et al., 2003; Horwood et al., 2006). PI3K has previously been shown to regulate AMPAR insertion during LTP, with inhibition of PI3K in neurons blocking LTP (Passafaro et al., 2001; Man et al., 2003). To test whether *Meg3* was able to modulate the PI3K/AKT signaling pathway in neurons, we first examined the phosphorylation level of the p85 regulatory subunit of PI3K at Tyr-458 as a proxy of PI3K activity during chem-LTP. In control neurons, glycine increased the level of phosphorylated p85 without altering the total protein expression (Figures 5A–C). In contrast, *Meg3* knockdown prevented the chem-LTP-dependent increase in the level of p85 phosphorylation (Figures 5A–C). Interestingly, the level of phosphorylated p85 was significantly higher under basal conditions in *Meg3* knockdown neurons than in control cells, which could prevent a further increase during chem-LTP (Figures 5A,B). Next, we analyzed the levels of AKT phosphorylation at Thr-308 and Ser-473, which are direct downstream targets of PI3K. We found that glycine induced a robust increase in the levels of phosphorylated AKT (Figures 5A,D,E) in control neurons. However, these increases were prevented in *Meg3* knockdown neurons (Figures 5A,D,E). Interestingly, the level of phospho-Thr-308 was significantly higher under basal conditions in *Meg3* knockdown neurons compared to control cells (Figures 5A,D). In contrast, the total AKT level was not affected by the reduction in *Meg3* expression (Figure 5F), ruling out translation as the mode of action. Consistent with previous findings, the loss of *Meg3* function caused partial overactivation of PI3K/AKT signaling, which correlates well with the higher accumulation of GluA1 on the plasma membrane under basal conditions. To test whether the overactivation of the PI3K/AKT signaling pathway was directly responsible for the increased level of surface GluA1 in *Meg3* knockdown neurons, we treated these neurons with two different PI3K inhibitors, wortmannin and LY294002. However, these treatments failed to rescue GluA1 surface expression in *Meg3* knockdown neurons (Supplementary Figure S5).

The cellular level of phosphatidylinositol (3,4,5)-triphosphate (PIP3) is tightly regulated by the lipid phosphatase, PTEN, which converts PIP3 into phosphatidylinositol (4,5)-bisphosphate (PIP2). This removes the localization of AKT from the plasma membrane, thereby counteracting the PI3K/AKT signaling pathway. Mutations that lead to PTEN loss of function have been associated with hyperactivity of AKT and its downstream target (Lugo et al., 2014). Intrigued by the failure of PI3K inhibitors to restore GluA1 surface expression in *Meg3* knockdown neurons, we next examined the level of PTEN protein expression in neurons. We found that the level of PTEN protein was significantly reduced in neurons following glycine stimulation (Figures 5A,G). Interestingly, PTEN expression was significantly downregulated in *Meg3* knockdown neurons and was no longer modulated by glycine (Figures 5A,G). In summary, our data demonstrate that *Meg3* is a critical regulator of the PTEN/PI3K/AKT signaling pathway and is crucial in modulating the number of GluA1-containing AMPARs on the neuronal plasma membrane.

## Discussion

LncRNAs constitute a vast proportion of the mammalian transcriptome and have recently emerged as critical regulators of many cellular and physiological functions. They are highly expressed in the brain and are known to play important roles in synaptogenesis and neuronal differentiation (Briggs et al., 2015). The expression of many lncRNAs is dynamically regulated by neuronal activity, making them prime candidates for controlling neuronal plasticity (Maag et al., 2015). However, there is currently no direct evidence demonstrating the involvement of lncRNAs in synaptic plasticity. In this study, we used the well-established chem-LTP protocol to induce NMDAR-dependent synaptic potentiation, and discovered a rapid induction of lncRNAs embedded within the *Dlk1-Dio3* locus in primary cortical neurons. The co-expression of these imprinted lncRNAs was striking, although it has been postulated that *Meg3* and the downstream lncRNAs may be transcribed as a large polycistronic unit (~200 kb) prior to further processing in some tissues due to the lack of well-defined promoters for the downstream genes (da Rocha et al., 2008). The binding of CREB to the cAMP response element located in the proximal region of the *Meg3* promoter may be responsible for the activation of *Meg3* transcription, and potentially that of other lncRNAs within the *Dlk1-Dio3* region during glycine stimulation (Zhao et al., 2006). Genetic deletion of the *Meg3* promoter impacts not only its own expression, but also that of the other downstream lncRNAs within the *Dlk1-Dio3* imprinted region (Zhou et al., 2010). Interestingly, the downstream lncRNAs, *Rtl1-AS* and *Meg9* harbor multiple clusters of miRNAs, many of which have been implicated in the pathophysiology of schizophrenia (Hagan et al., 2009; Gardiner et al., 2012). Although evidence of impaired LTP-like plasticity in patients with schizophrenia has been reported (Hasan et al., 2011; Frantseva et al., 2014), the molecular mechanisms underpinning this process are not well understood.

Using a cued-fear conditioning paradigm, we also found that the expression of a subset of the *Dlk1-Dio3* derived lncRNAs, including *Meg3*, *Meg8* and *Meg9*, was significantly upregulated in the hippocampus 2 h post-training. These data demonstrate that the expression of the *Dlk1-Dio3*-derived lncRNAs can also be dynamically regulated by associative learning *in vivo*. Although it is generally accepted that fear conditioning induces LTP (Rogan et al., 1997; DeAndrade et al., 2012), we currently do not have evidence to show the occurrence of NMDAR-dependent LTP in the hippocampi of mice that underwent this behavioral training. Notwithstanding that the learning-induced elevation in the expression levels of these lncRNAs is strictly dependent on the association between tones and shocks, we have not directly demonstrated the requirement of NMDAR activation in this process. Further experiments are required to establish the mechanisms that underlie the learning-induced expression of lncRNAs, as well as to determine their roles in the acquisition and/or recall of fear memory.

One of the major determinants of LTP is the number of AMPARs on the postsynaptic membrane, which is tightly regulated by multiple factors from their synthesis, endosomal trafficking, and endocytosis to the insertion onto the cell surface and their degradation rate (Anggono and Huganir, 2012). Previous studies have shown that certain miRNAs that target the 3′ untranslated region (UTR) of the *Gria1* and *Gria2* mRNAs, which encode the GluA1 and GluA2 subunits of AMPARs, respectively, are potent epigenetic regulators of LTP (miR-137: Olde Loohuis et al., 2015; miR-124: Gascon et al., 2014; Hou et al., 2015). However, the involvement of lncRNAs in regulating the surface expression of AMPARs in neurons is unknown. In this study, we focused solely on *Meg3* because it is expressed not only in the nucleus, but also in the cytoplasm, a feature that is unique among the *Dlk1-Dio3* lncRNAs. To determine whether *Meg3* plays a role in controlling the surface expression of the GluA1 subunit, we performed loss of function studies using two independent approaches, namely the shRNA- and ASO-mediated knockdown of *Meg3*. Surprisingly, we discovered that *Meg3* knockdown led to a significant increase in surface GluA1 expression concomitant with the overactivation of PI3K/AKT signaling. This pathway is known to play a crucial role in the exocytosis of AMPARs and LTP (Passafaro et al., 2001; Man et al., 2003; Opazo et al., 2003). Interestingly, *Meg3* knockdown only caused partial activation of AKT, as shown by an enhanced level of AKT phosphorylation at Thr-308 (a PDK1 site), but not at Ser-473 (an mTORC2 site). In order to test if the overactivation of PI3K was responsible for the basal increase in surface GluA1, we treated *Meg3* knockdown neurons with the PI3K inhibitors wortmannin and LY294002 for 1 h. However, the levels of surface GluA1 expression remained high. Given that the total protein level of PTEN is downregulated in *Meg3* knockdown neurons, it is plausible that a reciprocal increase in AKT signaling is still sufficient to enhance the expression of surface GluA1, even in the presence of PI3K inhibitors. Our data are consistent with previous studies which have demonstrated the potential role of *Meg3* as an endogenous competing RNA for a subset of miRNAs that regulate the level of PTEN in neurons (Peng et al., 2015; Yang et al., 2016). Accurate measurement of the strength and duration of PTEN/PI3K/AKT signaling will need to be investigated in a future study. This will include the determination of the levels of PIP3 in *Meg3* knockdown neurons.

The main finding of this study is that the loss of *Meg3* function blocks the NMDAR-dependent increase in surface GluA1 in primary cortical neurons. Due to the enhanced level of surface GluA1 in *Meg3* knockdown neurons, the activity-dependent increase in surface GluA1 expression seems to be occluded. However, we cannot rule out the possibility that the knockdown of *Meg3* leads to both an increase in surface AMPARs and a disturbance in AMPAR forward trafficking during glycine stimulation. In line with the latter hypothesis, we found that *Meg3* is indeed required for the full activation of AKT, which is necessary for AMPAR insertion onto the plasma membrane during glycine-induced synaptic potentiation. To further elucidate the role of *Meg3* in this activity-dependent AMPAR insertion, further experiments involving *Meg3* overexpression and rescue (in *Meg3* knockdown neurons) should be performed. The complexity of *Meg3* genomic structure (which yields multiple splice variants) and its large size (>10 kb) present challenges that preclude us from conducting these experiments in our current study (Schuster-Gossler et al., 1998; Hagan et al., 2009). Future experiments involving the use of the inducible dCAS9 system to transiently inhibit the transcription of *Meg3* would be ideal to decipher the role of *Meg3* in activity-dependent synaptic potentiation in neurons.

**Figure 6:**
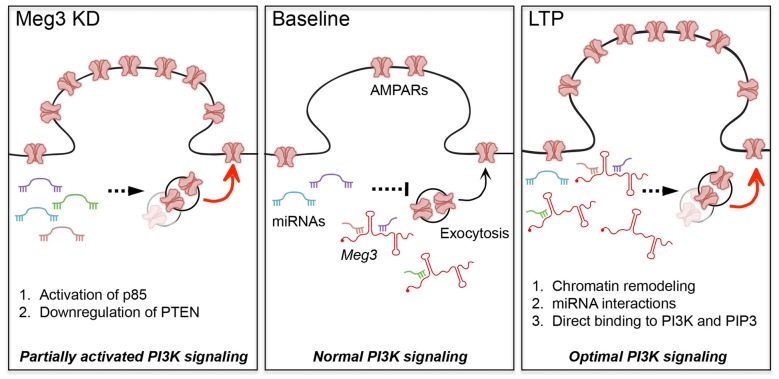
**Proposed models for the roles of *Meg3* in regulating surface GluA1 expression and activity-dependent synaptic potentiation.** *Meg3* has been proposed to act as an endogenous competing RNA for a subset of miRNAs that regulate the level of PTEN in neurons. We hypothesize that *Meg3* is involved in fine-tuning the activity of the PTEN/PI3K/AKT signaling pathway to regulate the optimal number of surface AMPARs in neurons. (Left panel) Downregulation of *Meg3* leads to a partial overactivation of AKT signaling via two distinct mechanisms, namely enhanced PI3K activity and the downregulation of PTEN expression, which subsequently lead to an increase in surface GluA1 expression. One of the underlying mechanisms might involve the loss of *Meg3* interactions with a subset of miRNAs that regulate essential components of the PTEN/PI3K/AKT signaling pathway. (Right panel) Our data suggest that *Meg3* is required for full activation of AKT and activity-dependent delivery of AMPARs to the plasma membrane during chem-LTP. We propose that *Meg3* may regulate synaptic plasticity through distinct regulatory mechanisms such as: (1) polycomb repressive complex 2 (PRC2)-dependent chromatin remodeling; (2) interaction with miRNAs; or (3) the allosteric modulation of PI3K and/or PIP3, ensuring the optimal activation of PI3K signaling to maintain LTP.

To reconcile our seemingly counterintuitive findings, in that *Meg3* knockdown enhances surface GluA1 and yet its expression is upregulated during chem-LTP, we propose the following working model (Figure 6). We hypothesize that the loss of *Meg3* function leads to a partial overactivation of AKT signaling via two distinct mechanisms, namely enhanced PI3K activity and the downregulation of PTEN expression, which subsequently lead to an increase in surface GluA1 expression. Based on emerging evidence in the literature, these effects are likely to be mediated by the loss of *Meg3* interactions with a subset of miRNAs that regulate essential components of the PTEN/PI3K/AKT signaling pathway (Peng et al., 2015; Pawar et al., 2016; Qiu et al., 2016; Yang et al., 2016). On the other hand, *Meg3* may regulate synaptic plasticity through distinct regulatory mechanisms such as: (a) an epigenetic mechanism via PRC2-dependent chromatin remodeling: (b) a post-transcriptional function through its interaction with miRNAs; or (c) the allosteric modulation of enzymes, including kinases, phosphatases or phospholipids. In the last of these mechanisms, it is plausible that *Meg3* could physically bind to the PI3K subunits (Koirala et al., 2017), PIP3 (Lin et al., 2017) or other downstream components of the PTEN/PI3K/AKT signaling pathway during LTP to ensure the correct activation of this pathway to maintain LTP. In summary, this study provides the first evidence demonstrating an involvement of the lncRNA *Meg3* in fine-tuning the activity of the PTEN/PI3K/AKT signaling pathway to regulate the optimal number of surface AMPARs in neurons during synaptic potentiation. Further studies are required to delineate the exact mechanisms by which *Meg3* and other lncRNAs contribute to synaptic plasticity, as well as the role of lncRNAs in adaptive behaviors.

## Author Contributions

MCT, JW, YQC, TZ and VA performed experiments. JW and VA designed research and wrote the manuscript. VA conceived the project and supervised the research. All authors analyzed the results and approved the final version of the manuscript.

## Conflict of Interest Statement

The authors declare that the research was conducted in the absence of any commercial or financial relationships that could be construed as a potential conflict of interest.

## Abbreviations

AMPARs: α-amino-3-hydroxy-5-methyl-4-isoxazolepropionic acid receptors
ASO: antisense oligonucleotide
CREB: cAMP response element binding protein
DIV: days *in vitro*
ERK: extracellular signal-regulated kinase
lncRNA: long non-coding RNA
LTP: long-term potentiation
*Meg*: maternally expressed gene
NMDA: *N*-methyl-*D*-aspartate
PI3K: phosphatidylinoside-3-kinase
PTEN: phosphatase and tensin homolog
*Rtl1-AS*: retrotransposon-like gene 1-antisense.
