# Supplementary material for: The Activity-Induced Long Non-Coding RNA Meg3 Modulates AMPA Receptor Surface Expression in Primary Cortical Neurons
Source: Front Cell Neurosci. 2017 May 3;11:124. doi: 10.3389/fncel.2017.00124 (PMC5413565; doi:10.3389/fncel.2017.00124)
Supplement: Supplementary file 1 [file Presentation_1.pdf]

Supplementary Material

The activity-induced long non-coding RNA *Meg3* modulates AMPA receptor surface expression in primary cortical neurons

Men Chee Tan[1,2†], Jocelyn Widagdo[1,2†*], Yu Qian Chau[1,2], Tianyi Zhu[1,2], Justin J.-L. Wong[3,4], Allen Cheung[2], Victor Anggono[1,2*]

[1]Clem Jones Centre for Ageing Dementia Research, The University of Queensland, Brisbane, QLD, Australia.

[2]Queensland Brain Institute, The University of Queensland, Brisbane, QLD, Australia.

[3]Gene & Stem Cell Therapy Program, Centenary Institute, Sydney, NSW, Australia

[4]Sydney Medical School, University of Sydney, Sydney, NSW, Australia

[†] These authors have contributed equally to this work.

*** Correspondence:**

Dr. Victor Anggono

v.anggono@uq.edu.au

Dr. Jocelyn Widagdo

j.widagdo@uq.edu.au

Supplementary Figures

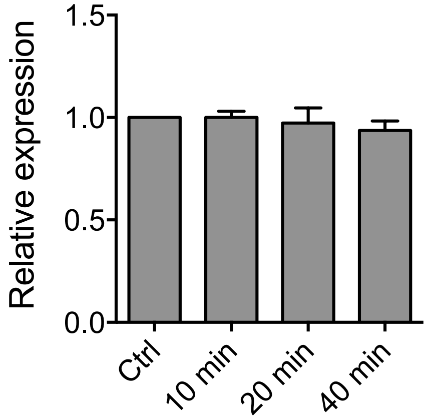

Supplementary Figure 1. The expression of *Gria2* mRNA following glycine stimulation. qPCR analyses of *Gria2* expression after normalization with *Gapdh*. Data represent the mean ± S.E.M. of three independent experiments (n = 3).

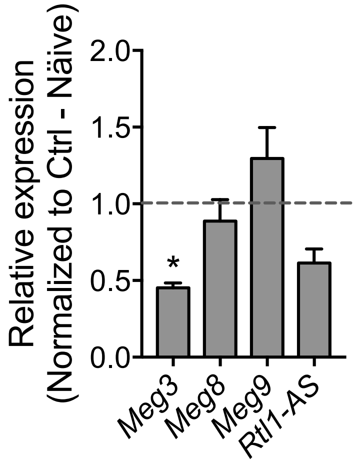

Supplementary Figure 2. The expression of *Dlk1-Dio3* imprinted lncRNAs in the mouse hippocampus 24 h post-fear conditioning. qPCR analyses of *Meg3*, *Meg8, Meg9* and *Rtl1-AS* expression after normalization with *Actb*. Data represent the mean ± S.E.M. (n = 4-5, unpaired *t*-test vs naïve controls, **P*<0.05).

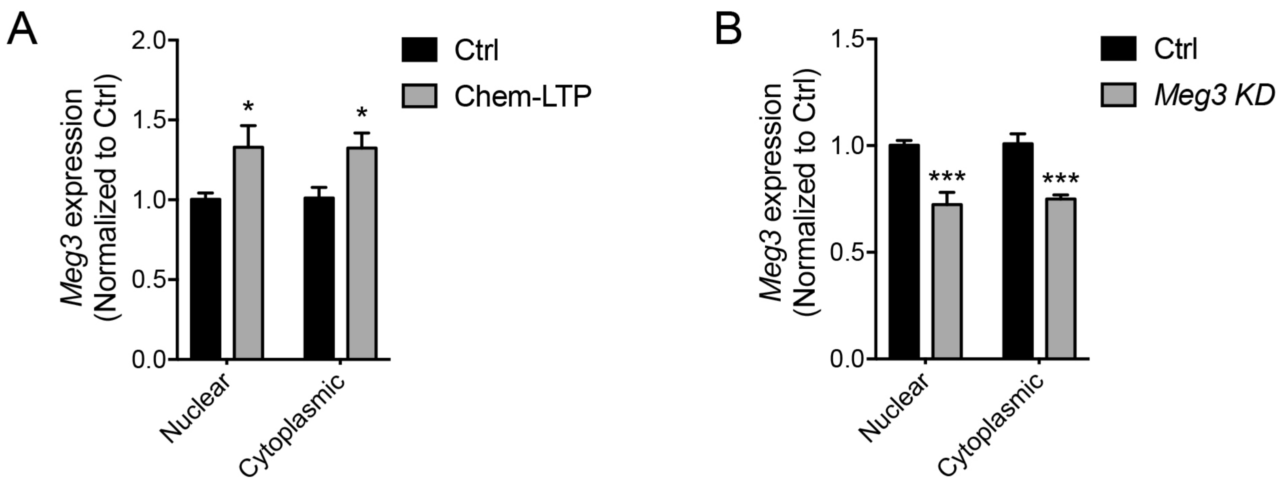

Supplementary Figure 3. Subcellular distribution of nuclear and cytoplasmic *Meg3*. qPCR analyses of *Meg3* expression in the nuclear and cytoplasmic fractions of cortical neurons that were **(A)** subjected to 10 min glycine stimulation (chem-LTP) or **(B)** transduced with FG12 (ctrl) or *Meg3* shRNA (*Meg3* KD) lentiviral particles, after normalization with *Actb*. Data represent the mean ± S.E.M. of three independent experiments (n = 6, unpaired *t*-test, *P<0.05, ***P<0.001).

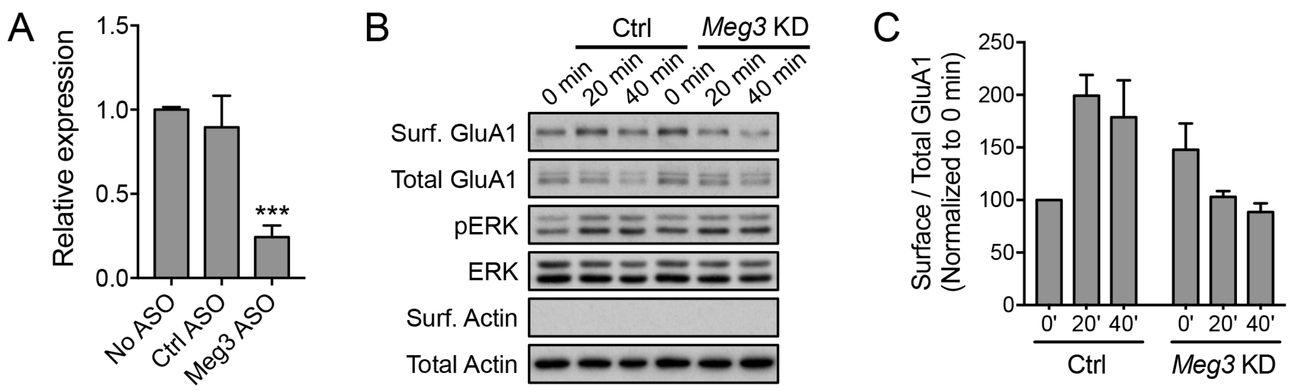

Supplementary Figure 4. Downregulation of *Meg3* disrupts AMPAR trafficking in primary neurons. (A) The efficiency of *Meg3* knockdown in cortical neurons treated with either control or *Meg3* ASOs was quantified by qPCR analysis (n = 3, unpaired *t*-test, ***P<0.001). **(B)** Surface biotinylation assay performed in *Meg3* knockdown and control neurons following chem-LTP. Representative western blots show the protein levels in the surface and total fractions. The absence of β-actin in the surface fractions was used to validate the biotinylation assay. **(C)** The effects of *Meg3* knockdown on the levels of surface and total GluA1 expression were quantified as surface/total receptor ratio and normalized to the 0 min time-point of the control cells. Data represent the mean ± S.E.M. of three independent experiments (n = 3).

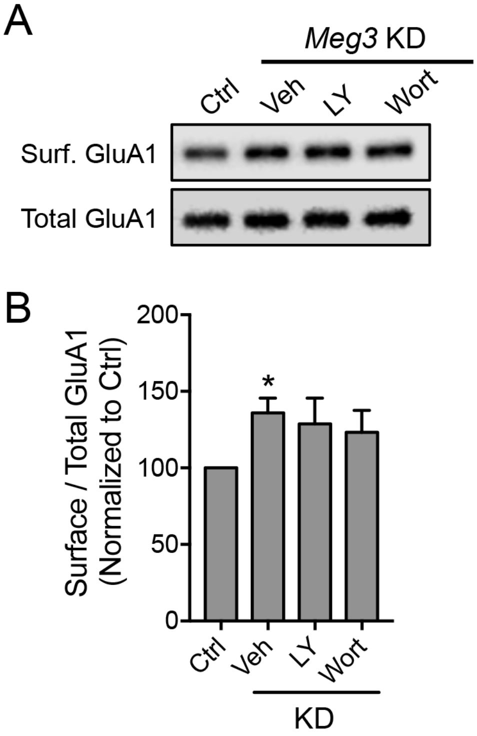

Supplementary Figure 5. The effects of PI3K inhibition on the levels of surface GluA1 expression in *Meg3* knockdown neurons. (A) Cortical neurons were transduced with either *Meg3* shRNA#2 (KD) or FG12 (ctrl) lentiviral particles at DIV8. At DIV13, *Meg3* knockdown neurons were incubated either DMSO (veh), 5 µM LY294002 (LY) or 200 nM wortmannin (Wort) for 1 h and subjected to protein biotinylation assay. Protein lysates were analyzed by western blotting. **(B)** Data represent the mean ± S.E.M. of three independent experiments (n = 3-5, one-way ANOVA, *P<0.05).
